# “Help” with HELLP syndrome

**DOI:** 10.1590/S1516-31802007000300014

**Published:** 2007-05-03

**Authors:** Krzysztof M. Kuczkowski

Pregnancy-induced hypertension (PIH), also known as preeclampsia, remains one of the leading causes of maternal death worldwide.^1^ The term PIH describes the development of hypertension with proteinuria and/or pathological edema after the twentieth week of gestation. Parturients with PIH usually have multiple organ alterations (Table 1). Although the etiology of PIH is unknown, it appears obvious that there is no single mechanism (factor) responsible for its development. Instead, several factors (mechanisms) have been implicated in the complex pathophysiology of this disease (Table 2).^1^

**Table 1.** Multiple organ involvement in pregnancy-induced hypertension

Cardiovascular changes
Hematological changes
Respiratory changes
Endocrine and metabolic changes
Hepatorenal changes
Neurological changes
Changes in uteroplacental blood flow

**Table 2.** Pathophysiology of pregnancy-induced hypertension

Immunological factors
Endothelial factors
Platelet factors
Coagulation factors
Calcium
Fatty acid metabolism

The HELLP syndrome is a pregnancy-specific disorder defined by hemolysis, elevated liver enzymes and low platelet count that is found in parturients with underlying PIH.^2^ The etiology of HELLP syndrome is unknown, and the pathogenesis of this disorder (including the hepatological manifestations) is not fully understood. Severe spontaneous bleeding into the liver parenchyma accompanied by hemorrhagic liver cell necrosis and rupture of the organ occurs in approximately 2% of patients with HELLP syndrome, and this represents the leading cause of death in these patients. Despite surgical interventions (based on trauma surgery principles), HELLP syndrome-associated liver rupture carries a mortality rate of 40-60%.^3^ Most patients die of hemorrhagic shock and/or multiple organ failure.^2,3^ Reck et al. recommended that, in order to improve maternal survival in cases of HELLP syndrome-associated hepatic rupture, an early interdisciplinary approach in a medical facility with extensive experience in liver surgery (including liver transplantation) is necessary.^3^ Postoperative care is equally challenging, with a propensity toward multiple systemic organ failure. However, with an aggressive multidisciplinary approach to the management of these patients, mortality rates may be decreased by 50%.^2–4^

It is with interest that I read the article by Osmanagaoglu et al.,^4^ published recently in the Sao Paulo Medical Journal, which highlights an important learning point regarding peripartum care for pregnant women with HELLP syndrome: the need for an aggressive multidisciplinary approach and prompt transfer of these women to obstetric centers with expertise in this field.

The author of the present communication (together with a multispecialty team of experts) at the University of California, San Diego, provided subspecialty care for an otherwise healthy female at 32 weeks of gestation that was complicated by PIH and HELLP syndrome. She was transferred to our tertiary care University Hospital from a smaller local hospital with hepatic rupture following a minor motor vehicle accident.^1^

On admission to the local hospital she was tachycardic and hypotensive, and complained of an intensifying right upper quadrant abdominal pain (without any obvious injuries). Laboratory analysis revealed hematocrit of 28%, platelet count of 49,000/mm^3^, serum aspartate aminotransferase (AST) of 1771 U/liter and serum alanine aminotransferase (ALT) of 1367 U/liter (normal AST and ALT levels are lower than 35-45 U/liter). Cesarean section under general anesthesia (rapid sequence induction with cricoid pressure) was performed because of persistent fetal bradycardia. Following delivery of a healthy newborn with good Apgar scores, a large amount of free intraperitoneal blood was encountered. In view of the ongoing bleeding (but with adequate blood and fluid replacement therapy), the patient's abdomen was surgically explored. A right hepatic lobe laceration extending horizontally from the inferior lateral edge of the liver to the falciform ligament, with concomitant subcapsular hematoma extending from the laceration superiorly to the dome of the liver, was noted. Perihepatic packing was performed, two perihepatic drains were placed, and the patient was transferred as an emergency case to the University of California Medical Center, where angiographic hepatic embolization and argon beam photocoagulation of the laceration were performed and hemostasis was achieved. Her postoperative course was marked by severe and persistent hypertension, renal failure (serum creatinine of 5.5 mg/dl) necessitating hemodialysis, and prolonged mechanical ventilation requiring tracheotomy. Following a six-week period of convalescence, she achieved a full recovery.^1^

In conclusion, the good outcome in this report seems to illustrate very well the point raised by Osmanagaoglu et al.^4^ regarding early diagnosis and timely transfer (an early call for help) for patients with HELLP syndrome, to obstetric departments with expertise in this field.
